# Ranking of persister genes in the same *Escherichia coli* genetic background demonstrates varying importance of individual persister genes in tolerance to different antibiotics

**DOI:** 10.3389/fmicb.2015.01003

**Published:** 2015-09-30

**Authors:** Nan Wu, Lei He, Peng Cui, Wenjie Wang, Youhua Yuan, Shuang Liu, Tao Xu, Shanshan Zhang, Jing Wu, Wenhong Zhang, Ying Zhang

**Affiliations:** ^1^Key Lab of Molecular Virology, Institute of Medical Microbiology, Department of Infectious Diseases, Huashan Hospital, Fudan UniversityShanghai, China; ^2^Department of Molecular Microbiology and Immunology, Bloomberg School of Public Health, Johns Hopkins UniversityBaltimore, MD, USA

**Keywords:** *Escherichia coli*, persistence, persister gene, knockout mutant, antibiotics, ranking

## Abstract

Despite the identification of many genes and pathways involved in the persistence phenomenon of bacteria, the relative importance of these genes in a single organism remains unclear. Here, using *Escherichia coli* as a model, we generated mutants of 21 known candidate persister genes and compared the relative importance of these mutants in persistence to various antibiotics (ampicillin, gentamicin, norfloxacin, and trimethoprim) at different times. We found that *oxyR*, *dnaK*, *sucB*, *relA*, *rpoS*, *clpB*, *mqsR*, and *recA* were prominent persister genes involved in persistence to multiple antibiotics. These genes map to the following pathways: antioxidative defense pathway (*oxyR*), global regulators (*dnaK*, *clpB*, and *rpoS*), energy production (*sucB*), stringent response (*relA*), toxin–antitoxin (TA) module (*mqsR*), and SOS response (*recA*). Among the TA modules, the ranking order was *mqsR*, *lon*, *relE*, *tisAB*, *hipA*, and *dinJ.* Intriguingly, *rpoS* deletion caused a defect in persistence to gentamicin but increased persistence to ampicillin and norfloxacin. Mutants demonstrated dramatic differences in persistence to different antibiotics at different time points: some mutants (*oxyR*, *dnaK*, *phoU*, *lon*, *recA*, *mqsR*, and *tisAB*) displayed defect in persistence from early time points, while other mutants (*relE*, *smpB*, *glpD*, *umuD*, and *tnaA*) showed defect only at later time points. These results indicate that varying hierarchy and importance of persister genes exist and that persister genes can be divided into those involved in shallow persistence and those involved in deep persistence. Our findings suggest that the persistence phenomenon is a dynamic process with different persister genes playing roles of variable significance at different times. These findings have implications for improved understanding of persistence phenomenon and developing new drugs targeting persisters for more effective cure of persistent infections.

## Introduction

Persisters are a small subpopulation of generally quiescent bacterial cells that are tolerant to bactericidal antibiotics (Lewis, 2010). In contrast to resistant cells, persisters are phenotypically and genetically identical to susceptible bacteria (Balaban et al., 2004). Studies have shown that persisters play a role in treatment failure in persistent infections such as tuberculosis (Zhang et al., 2012), urinary tract infections (Blango and Mulvey, 2010), and biofilm infections (Singh et al., 2009; Levin et al., 2014), underscoring the need for improved understanding of bacterial persistence and better treatment. Although the phenomenon of bacterial persistence was discovered over 70 years ago (Hobby et al., 1942; Bigger, 1944), it is only recently that researchers began to understand the mechanisms of persister formation.

A number of genes and pathways have been found to be associated with persister formation or survival. Since the first toxin protein HipA was linked to persistence in *Escherichia coli* in Moyed and Bertrand (1983), increasing evidence suggests that persistence is only partially attributed to the toxin–antitoxin (TA) modules (Lewis, 2010). Other genes involved in persistence are found in the pathways of stringent response (Korch et al., 2003), SOS response (Debbia et al., 2001; Dorr et al., 2009), energy metabolism (Ma et al., 2010; Girgis et al., 2012), global regulators such as PhoU (Li and Zhang, 2007), trans-translation (Shi et al., 2011; Li et al., 2013) and signaling pathways (Vega et al., 2012). These findings suggest that persistence is a very complex phenomenon with redundant mechanisms. Despite the above progress, the studies that led to the identification of various persister genes were performed in different strains, or with the same strain but one antibiotic or one time point, or by different investigators under different conditions. Thus, the relative importance of the identified persister genes and pathways in persister formation is unknown and has never been evaluated in a single study under the same conditions in the same strain over different time points. We hypothesize that not all persister genes are created equal and that different persister genes may play a different role under different conditions.

In the present study, we assessed whether some persister genes or pathways play more important roles than others in conferring the persistence phenotype. Taking advantage of the convenient genetic manipulation of the model organism *E. coli*, we constructed deletion mutants of 21 known persister genes (Zhang, 2014) and ranked their relative importance in persistence in different antibiotic exposure assays. Our data revealed varying degrees of decreased persistence among different mutants. They also showed that different persistence genes have a different role with variable importance in persistence at different times. Our findings provide valuable information on bacterial persistence genes and shed new light on the complexity of the persistence phenomenon.

## Materials and Methods

### Bacterial Strains and Growth Media

The *E. coli* K12 W3110 bacterial strain used in this work is the wild-type (F^-^*mcrAmcrB* IN(*rrnD-rrnE*)*1* lambda^-^), and was used for construction of knockout mutants of 21 persister genes. Luria–Bertani (LB; 0.5% NaCl) broth and agar (15 g/L) were used for routine cultivation of the *E. coli* strains. To ensure the reproducibility of the results, LB medium was prepared by filter sterilization rather than by autoclaving.

### Construction of *E. coli* W3110 Knockout Mutants

Disruption of 21 candidate persister genes in the *E. coli* chromosome was achieved by using the λ Red recombination system, as previously described by Datsenko and Wanner (2000). Further details of primers designed for this purpose and additional external primers used to verify the correct integration of the PCR fragments by homologous recombination are shown in Supplementary Table S1.

### Persister Assay

Persistence was measured by determining bacterial survival in the form of colony forming units upon exposure to four antibiotics, namely, ampicillin at 100 μg/ml; norfloxacin at 4 μg/ml; gentamicin at 20 μg/ml; and trimethoprim at 64 μg/ml (Li et al., 2013). A single type of antibiotic was used for each knockout gene mutant. *E. coli* cells were grown to stationary phase in LB medium, and 1:100 dilutions (Luidalepp et al., 2011) were made in fresh medium containing a specific antibiotic. The antibiotic exposure was carried out over a period of several hours to 7 days at 37°C without shaking. Samples were withdrawn at the indicated times, diluted in sterile saline, and plated on LB agar without antibiotics. Colonies were counted on the following day after overnight incubation at 37°C.

### Susceptibility of Mutants to Various Antibiotics

Minimum inhibitory concentration (MIC) and minimum bactericidal concentrations (MBC) of ampicillin, gentamicin, norfloxacin, and trimethoprim were determined by using serial twofold dilutions of the antibiotics in LB broth. The bacterial inocula consisted of 10^6^ to 10^7^ bacteria/ml of diluted stationary-phase cultures, and the samples were incubated for 18 h at 37°C without shaking. The MIC was recorded as the minimum drug concentration that prevented visible growth, and the MBC was recorded as the lowest concentration that killed 99.9% of the initial inoculum.

### Persister Gene Scoring

Persister genes were ranked according to cell survival under exposure to four different antibiotics at different times. The persisters genes whose mutants showed significant difference compared to the parent strain were scored as “1” point, whereas those mutants that did not show significant difference compared to the parent strain were scored as “0”. All scores for a given mutant were calculated from the sum of values from different antibiotic exposures to obtain the ranking of the persister genes (**Table 1**; Supplementary Tables S2–S4).

**Table 1:** Comprehensive ranking of persister genes and pathways according to scores^∗^.

	Mutated persister genes	Persistence pathways	KEGG pathways	Score
1	*oxyR*	Antioxidant defense		10
2	*dnaK*	Global regulator	RNA degradation	9
3	*sucB*	Energy production	Lysine degradation| Citrate cycle (TCA cycle)| Carbon metabolism	8
	*relA*	Stringent response	Purine metabolism	8
4	*rpoS*	Global regulator		7
5	*clpB*	Global regulator		6
	*mqsR*	TA module		6
	*recA*	SOS response	Homologous recombination	6
6	*lon*	TA module/protease		5
7	*phoU*	Global regulator		4
	*smpB*	Trans-translation		4
	*glpD*	Energy production	Glycerophospholipid metabolism	4
	*relE*	TA module		4
8	*ssrA*	Trans-translation		3
	*uvrA*	SOS response	Nucleotide excision repair	3
	*tisAB*	TA module		3
9	*tnaA*	Signaling pathway	Tryptophan metabolism	1
	*umuD*	SOS response		1
	*hipA*	TA module		1
10	*pspF*	Signaling pathway		0
	*dinJ*	TA module		0

*^∗^Persister genes were ranked according to cell survival under exposure to four different antibiotics at different times. The persisters genes whose mutants show significant difference compared to the parent strain were scored as “1” point, whereas those mutants that did show significant difference were scored as “0”. All scores for a given mutant are calculated from the sum of different antibiotic exposures to obtain the ranking order of the 21 persister genes.*

### Statistical Analysis of Data

The statistical significance of the data (wild-type versus mutant upon exposure to a specific antibiotic) was evaluated with non-parametric tests (Mann–Whitney *U* tests). Data are representative of three independent experiments. All data are presented as the mean ± SD. A *P*-value <0.05 was considered statistically significant.

## Results

### Susceptibility of Deletion Mutants of Known Persister Genes to Various Antibiotics

**FIGURE 1:**
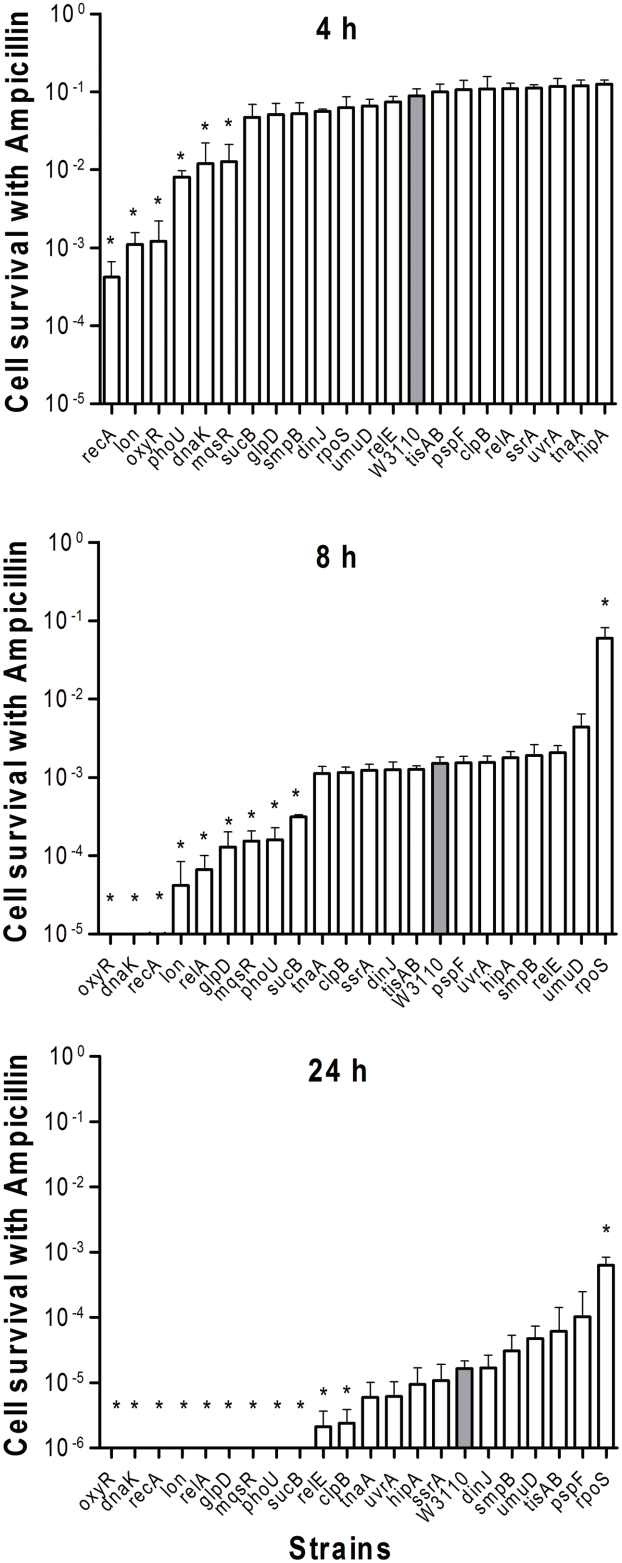
**Deletion mutant ranking depends on cell survival over time under conditions of ampicillin exposure.** Stationary phase cultures of W3110 (gray bars) and single-gene mutants (white bars) were diluted 100-fold and exposed to 100 μg/ml ampicillin for 4, 8, and 24 h. The data for each mutant was plotted and compared with that of W3110. Error bars indicate the standard deviation (*n* = 3). The asterisk indicates statistical significance as determined using Mann–Whitney *U* tests (^∗^*P* < 0.05).

To determine the persister levels for the persister gene deletion mutants, the stationary phase cultures of the mutants and the wild-type strain W3110 were exposed to the following four antibiotics, respectively, including ampicillin (100 μg/ml), norfloxacin (4 μg/ml), gentamicin (20 μg/ml), and trimethoprim (64 μg/ml). The survival of the mutants at different time points under exposure of a single antibiotic was assessed. The results showed that about half the mutants were more susceptible to most antibiotics than the parent strain W3110. Upon treatment with ampicillin, the persister levels of most knockout strains decreased significantly at 2 h, while those of the wild-type strain decreased from 4 h antibiotic exposure (data not shown). It is worth noting that persister levels in *oxyR*, *dnaK*, *recA*, *lon*, *relA*, *glpD*, *mqsR*, *phoU*, and *sucB* mutants were below the limit of detection (10 CFU/ml) after ampicillin exposure after 1 day, whereas about three orders of magnitude (0.01%) of the parent control strain W3110 cells still remained (**Figure 1**). The persister gene mutants were ranked from 4 h, because almost all the mutants demonstrated the same magnitude of decrease before this time point. The results showed that deletion of *recA*, *lon*, *oxyR*, *phoU*, *dnaK*, and *mqsR* significantly decreased persister formation (>6.9-fold) compared with W3110. At 8 h, in addition to the six genes above, the *relA*, *sucB*, and *glpD* mutants also displayed a dramatic decrease in persister levels. Furthermore, the *relE* and *clpB* mutants showed a significant decrease at 24 h. Therefore, the 11 genes could be divided into three groups according to the time points when a significant defect in persistence was observed, suggesting that different persister genes play roles of variable importance at different time points during the persistence phenomenon.

**FIGURE 2:**
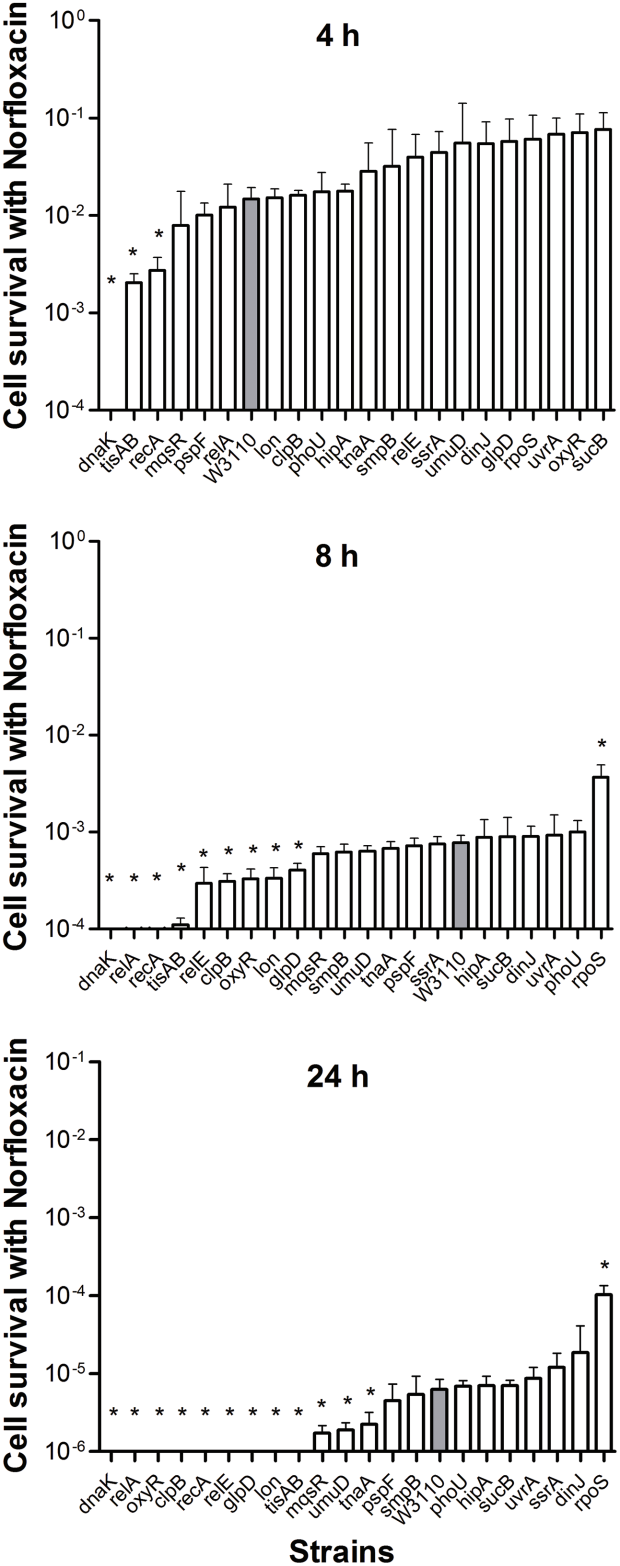
**Deletion mutant ranking depends on cell survival over time under conditions of norfloxacin exposure.** Stationary phase cultures of W3110 and single-gene mutants were diluted 100-fold and exposed to 4 μg/ml norfloxacin for 4, 8, and 24 h. The data for each mutant was plotted and compared with that of W3110. Error bars indicate the standard deviation (*n* = 3). The asterisk indicates statistical significance as determined using Mann–Whitney *U* tests (^∗^*P* < 0.05).

The pattern of results from the norfloxacin treatment was similar to that obtained using ampicillin. Cells were progressively killed during an 8 h period. The decrease in persister levels was observed from 2 h after norfloxacin treatment and no surviving bacteria were detected in nine mutants (*dnaK*, *relA*, *oxyR*, *clpB*, *recA*, *relE*, *glpD*, *lon*, and *tisAB*) at 24 h (**Figure 2**), whereas the parent strain had 2 × 10^2^ viable bacteria left.

The persister gene ranking was initiated at 4 h owing to the same reason mentioned above. As shown in **Figure 2**, only Δ*dnaK*, Δ*tisAB* and Δ*recA* mutants showed more than 5.4-fold decrease in persister cell survival at 4 h. Moreover, the persister cell survival in Δ*dnaK* was found to drop sharply even from the first 2 h. Other two mutants (*tisAB, recA*) demonstrated significant decrease in persister levels from 4 h (**Figure 2**). At 24 h, the other nine mutants (*relA*, *relE*, *clpB*, *oxyR*, *lon*, *glpD*, *mqsR*, *umuD*, and *tnaA*) exhibited a dramatic decrease by more than 2.7-fold compared with the wild-type (**Figure 2**). These nine genes (*relA*, *oxyR*, *clpB*, *relE*, *glpD*, *lon*, *mqsR*, *umuD*, and *tnaA*) compared to *dnaK*, *recA*, *tisAB* could be long-time stress dependent genes, and may be called ‘deep persister’ genes to quinolone stress. From our data, *dnaK* showed the highest sensitivity to norfloxacin among the 21 mutants with the shortest survival time, suggesting the importance of *dnaK* in persister cell formation under stress of fluoroquinolone antibiotics. Other genes, including *relA*, *clpB*, *glpD*, *relE*, *oxyR*, *lon*, etc., may support persistence later, from 8 h (**Figure 2**).

**FIGURE 3:**
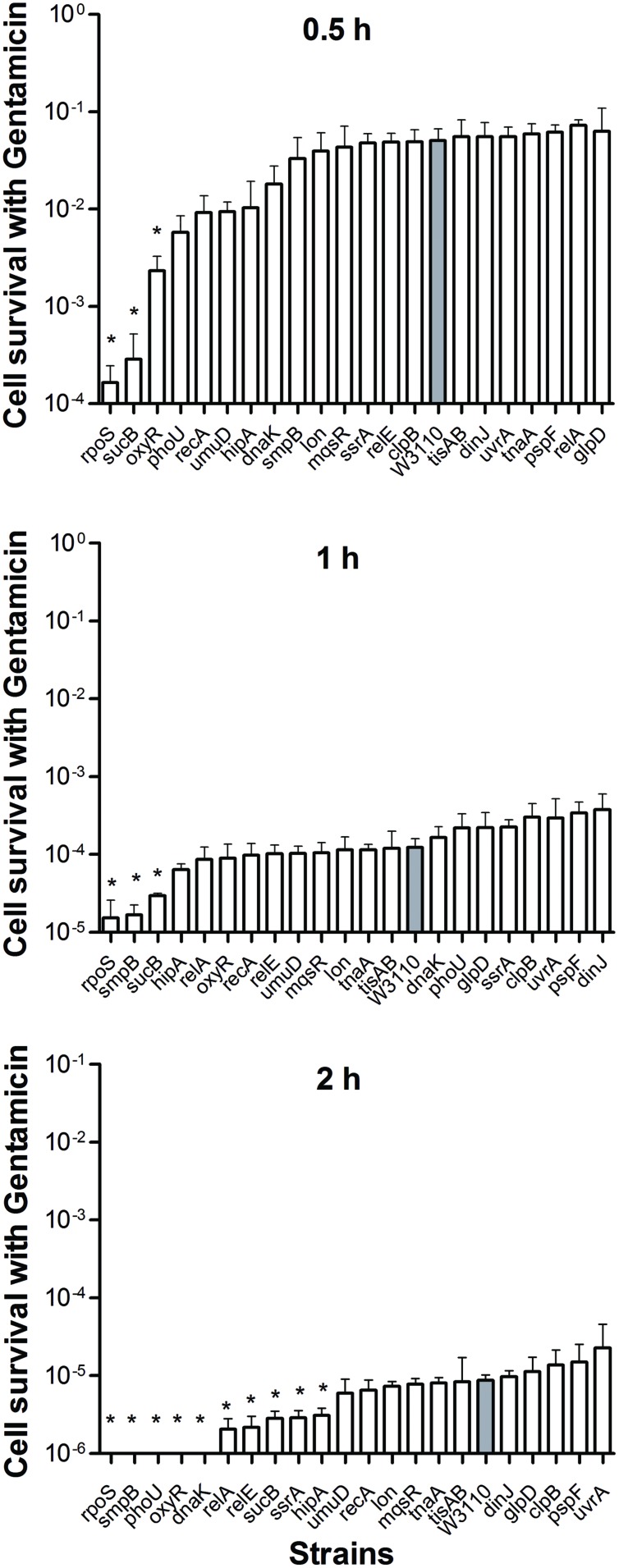
**Deletion mutant ranking depends on cell survival over time under conditions of gentamicin exposure.** Stationary phase cultures of W3110 and single-gene mutants were diluted 100-fold and exposed to 20 μg/ml gentamicin for 0.5, 1, and 2 h. The data for each mutant was plotted and compared with that of W3110. Error bars indicate the standard deviation (*n* = 3). The asterisk indicates statistical significance as determined using Mann–Whitney *U* tests (^∗^*P* < 0.05).

When stationary phase cells were transferred to fresh medium, considerably fewer gentamicin-tolerant persisters were alive after 4 h. All of the mutant cells were completely killed by gentamicin in 8 h including the wild-type strain (data not shown). Given the rapid sterilization by gentamicin, we ranked these mutants 0.5 h onward. After 2 h treatment, gentamicin effectively sterilized *rpoS*, *smpB*, *phoU*, *oxyR*, and *dnaK* mutants and the number of CFU dropped below the detection limit, whereas the wild-type strain had about 2.3 × 10^2^ (0.001%) viable bacteria left. Interestingly, unlike the high persistence during ampicillin and norfloxacin treatment, we found that deletion of *rpoS* caused a significant decrease in persister levels when the mutant was subjected to gentamicin compared with the parent strain (**Figure 3**).

**FIGURE 4:**
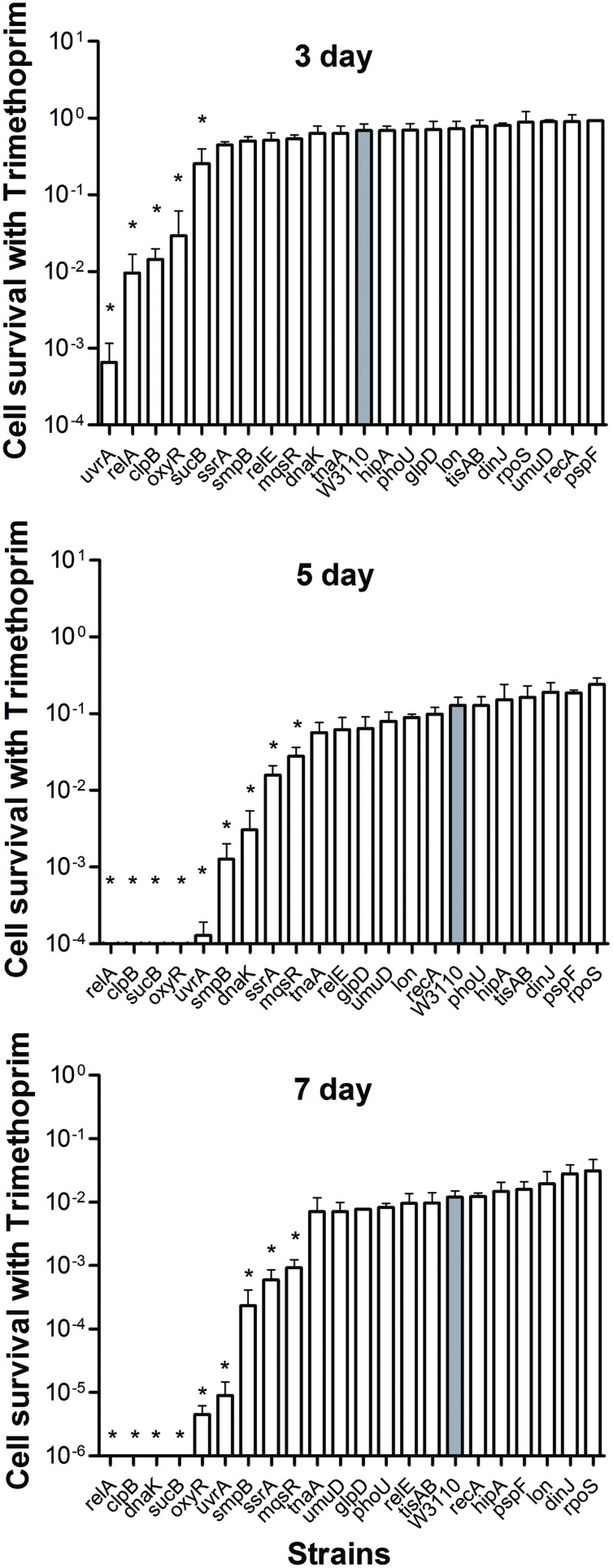
**Deletion mutant ranking depends on cell survival over time under conditions of trimtheprim exposure.** Stationary phase cultures of W3110 and single-gene mutants were diluted 100-fold and exposed to 64 μg/ml trimethoprim for 3, 5, and 7 days. The data for each mutant was plotted and compared with that of W3110. Error bars indicate the standard deviation (*n* = 3). The asterisk indicates statistical significance as determined using Mann–Whitney *U* tests (^∗^*P* < 0.05).

Both mutants and the wild-type strain showed higher tolerance to the bacteriostatic agent trimethoprim. After exposure to trimethoprim, the strains showed a lower decrease in colony counts compared with the other three bactericidal antibiotics. Interestingly, trimethoprim becomes bactericidal to some mutants during prolonged incubation (see below). The *uvrA*, *relA*, *clpB*, *oxyR*, and *sucB* deletion mutants had significantly lower persister levels from day 3, when the wild-type strain still had a high percentage of persisters at 68.8% (**Figure 4**). At day 5, other genes, s*mpB*, *dnaK*, *ssrA*, and *mqsR* mutants entered into the dramatically decreasing phase. In contrast to the *clpB* and *relA* mutants, where reduction was almost top ranked through days 3–7, the decreasing trend of the *uvrA* mutant slowed from the topmost position at day 3 and was replaced by other mutants at the following time points (**Figure 4**). The data again support the notion that the importance of persister genes is relative, and varies with time (Li and Zhang, 2007; Ma et al., 2010). Moreover, *relA*, *clpB*, *dnaK*, and *sucB* mutant strains were more susceptible than the wild-type strain to the bacteriostatic antibiotic trimethoprim such that no viable bacteria were left in these mutants after 7 days exposure, while the wild-type strain had about 3.3 × 10^5^ CFU/ml remaining.

### Ranking of Persister Genes According to Pathways in Persister Formation

We were able to rank the 21 persister genes into 10 grades or levels according to the ranking scores calculated based on difference of the mutants from the parent strain in response to different antibiotics over different time points (**Table 1**). The genes belonging to TA modules, global regulators, stringent response, energy production, and signaling pathways showed lower persistence levels and higher scores in multiple antibiotic exposure assays (**Table 1**; Supplementary Tables S2–S4). Many studies suggested that TA modules may be involved in persister formation (Keren et al., 2004; Buts et al., 2005; Vazquez-Laslop et al., 2006; Wang and Wood, 2011; Hong et al., 2012; Kaspy et al., 2013; Wang et al., 2013). Persistence can be induced by overexpression of various toxins: HipA, RelE, MqsR and MazF (Falla and Chopra, 1998; Kim et al., 2010; Maisonneuve et al., 2011; Tashiro et al., 2012). In this study, six TA module genes (*relE*, *hipA*, *mqsR*, *tisAB*, *lon*, and *dinJ*) were used to determine which genes are more important in this pathway. Among the six selected TA gene mutants, *mqsR* and *relE* mutants demonstrated the most obvious persistence defect in being susceptible to three antibiotics tested (ampicillin, norfloxacin, and trimethoprim for *mqsR* mutant, ampicillin, norfloxacin, and gentamicin for *relE* mutant; **Figures 1–4**), while other four TA genes (*hipA*, *tisAB*, *lon*, and *dinJ*) showed persistence defect with exposure to one or two antibiotics (**Figures 1–4**), indicating the four genes (*hipA*, *tisAB*, *lon*, and *dinJ*) are involved in narrower range of antibiotic tolerance compared to *mqsR* and *relE*. It was worth noting that, although *lon* mutant was only sensitive to ampicillin and norfloxacin, it was still an important TA gene according to its higher score compare with that of *relE* mutant. These results suggest that different elements of the same pathway (or similar molecular function/biological process) can display diverse responses toward different antibiotic stress and the importance of different genes in a specific persistence pathway can vary. Our findings suggest *mqsR*, *lon*, and *relE* may be the more important genes in persistence among the six TA modules tested.

We also observed varying importance of persister genes in other pathways. SOS response is an inducible DNA repair system that is initiated when RecA senses damaged DNA and promotes cleavage of the global repressor LexA (Michel, 2005). In the pathway of SOS response, *recA* mutant showed more obvious defect than *umuD* and *uvrA* mutants in persister formation under bactericidal antibiotic exposure (**Table 1**). The bacterial stationary-phase signaling molecule indole may act through antioxidant defense OxyR and phage-shock pathways to induce persister formation in *E. coli* (Vega et al., 2012). Among the genes of these different signaling pathways, the *oxyR* mutant had persister defect during exposure to all four antibiotics from early timepoints and was the top gene due to its highest score (**Table 1**), whereas *pspF* and *tnaA* mutants showed defects only when exposed to norfloxacin at 24 h (**Figures 1–4**).

In the group of global regulators (Hansen et al., 2008; Matsuoka and Shimizu, 2011), three genes, *dnaK*, *clpB*, and *phoU* were studied. DnaK and ClpB belong to a family of Heat shock protein, which are molecular chaperones that cooperate in the chaperone-mediated, ATP-dependent unfolding of protein aggregates (Straus et al., 1990; Szabo et al., 1994), and also act as regulators of a large series of genes induced by heat shock and general stress response (Tilly et al., 1983; Squires et al., 1991; Muﬄer et al., 1996). It has been suggested that DnaK may be required for the maintenance of persistence, as a *dnaK* deleted strain produced decreased number of persisters (Singh et al., 2007; Hansen et al., 2008). In this study, *clpB* and *dnaK* mutants had defect in persister formation in exposure to three and four antibiotics, respectively, indicating the importance of global regulators for persister cells (**Table 1**). Our results showed that *dnaK* not only was more important than *clpB* in global regulators, but also played a crucial role, comparable in degree to genes in other pathways, such as *oxyR* and *relA* in persister formation, both of which demonstrated importance in exposure to four antibiotics. PhoU, whose expression is regulated by environmental changes like nutrient availability and age of culture, is a global negative regulator beyond its role in phosphate metabolism (Li and Zhang, 2007). In this study, *phoU* mutant had a dramatic defect in persister phenotype with ampicillin and gentamicin (**Figures 1** and **3**), consistent with our previous findings (Li and Zhang, 2007).

*sucB and glpD* are involved in energy metabolism pathways, and have been shown to play a role in persister formation and tolerance to multiple antibiotics and stresses in *E. coli* (Yeh et al., 2008; Ma et al., 2010). Both *sucB* and *glpD* mutants were more susceptible to ampicillin during prolonged exposures (24 h) but they did not have an obvious decrease in persistence compared to that during a short exposure of 4 h (**Figure 1**).

### Deletion of *rpoS* Increased Persistence to Ampicillin and Norflaxacin Treatment

In the present study, almost all mutants showed varying degrees of defects in persistence to antibiotics, except for the *rpoS* mutant. Sigma factor RpoS regulation is one of the major stress resistance mechanisms in bacteria in the stationary phase since RpoS regulates many stress-responsive genes (Hengge-Aronis, 1996; Gerard et al., 1999). Paradoxically, cells that lack *rpoS* dramatically increased persister production compared with the parent strain during ampicillin treatment (8 and 24 h) (**Figure 1**). Furthermore, deletion of *rpoS* significantly increased persister production (about 17-fold) compared with the parent strain during exposure to norflorxacin (4 μg/ml) exposure, such that 0.01% of the cells were present in the *rpoS* mutant while the parent strain had only 0.0006% viable persister cells remaining after exposure to norflorxacin for 24 h (**Figure 2**). However, the *rpoS* mutant was more susceptible to gentamicin exposure than the parent strain (**Figure 3**).

### Minimum Inhibitory Concentration and Minimum Bactericidal Concentrations of the Persister Gene Deletion Mutants

Although it is generally assumed that persister genes have no effect on the MIC, some mutants defective in persister genes, such as *phoU*, *sucB*, and *smpB* may exhibit a slightly increased sensitivity to antibiotics (Li and Zhang, 2007; Ma et al., 2010; Li et al., 2013). In order to assess if this is a more general phenomenon among persister genes, we determined the susceptibility of all the 21 persister gene mutants to a variety of antibiotics in MIC and MBC tests. In general, there were 2- to 4-fold changes between all mutants and the wild-type strain to three bactericidal antibiotics ampicillin, gentamicin, and norfloxacin (**Table 2**). An interesting observation is that many persister gene mutants were found to be more susceptible to the sulfa drug trimethoprim than wild-type W3110 in both MIC and MBC tests (**Table 2**).

**Table 2:** Minimum inhibitory concentration (MIC) and minimum bactericidal concentration (MBC) determination for 21 persister mutants and the parent strain *E. coli* W3110 using different antibiotics^∗^.

MIC/MBC (mg/L)				
Strains	Ampicillin	Gentamicin	Norfloxacin	Trimethoprim
Δ*glpD*	6.25/25	2.5/2.5	0.125/0.125	0.5/2
Δ*relA*	6.25/12.5	1.25/1.25	0.125/0.125	0.5/2
Δ*uvrA*	12.5/25	1.25/2.5	0.125/0.125	0.5/2
Δ*umuD*	6.25/12.5	2.5/5	0.25/0.5	0.25/1
Δ*lon*	6.25/12.5	2.5/2.5	0.25/0.25	0.5/4
Δ*relE*	6.25/12.5	2.5/2.5	0.125/0.125	0.125/0.5
Δ*smpB*	6.25/6.25	1.25/2.5	0.06/0.25	0.125/0.25
Δ*ssrA*	6.25/6.25	2.5/2.5	0.06/0.25	0.25/1
Δ*dinJ*	12.5/25	2.5/5	0.125/0.125	0.5/2
Δ*rpoS*	12.5/25	1.25/1.25	0.25/0.25	0.5/4
Δ*tnaA*	6.25/12.5	2.5/2.5	0.125/0.125	0.5/1
Δ*pspF*	12.5/25	2.5/2.5	0.125/0.125	0.5/2
Δ*mqsR*	6.25/12.5	2.5/2.5	0.25/0.25	0.5/2
Δ*clpB*	12.5/25	1.25/2.5	0.125/0.25	0.06/0.25
Δ*phoU*	3.13/6.25	1.25/1.25	0.06/0.125	0.25/1
Δ*hipA*	6.25/6.25	1.25/1.25	0.125/0.125	0.25/1
Δ*recA*	6.25/12.5	1.25/2.5	0.125/0.125	0.25/2
Δ*tisAB*	3.13/6.25	1.25/1.25	0.125/0.125	0.25/1
Δ*sucB*	6.25/12.5	1.25/2.5	0.06/0.125	0.25/2
Δ*oxyR*	12.5/25	2.5/2.5	0.25/0.25	0.25/1
Δ*dnaK*	6.25/12.5	1.25/1.25	0.25/0.5	0.125/0.5
W3110	6.25/12.5	2.5/2.5	0.125/0.125	0.5/2

*^∗^The first value is MIC and the second value under ‘/’ refers to MBC.*

## Discussion

Although a significant number of persister genes have been identified (Kint et al., 2012; Amato et al., 2014), they were identified in different studies under different conditions and their relative importance in bacterial persistence in one single study under the same conditions in the same genetic background using varying timepoints have not been performed. Here, using *E. coli* as a model, we newly constructed deletion mutants of 21 candidate persister genes and analyzed the relative importance of these persiser genes or pathways with different antibiotics over time. While *E. coli* KEIO library was used in a single genetic background under a single condition, it was done only with one antibiotic ofloxacin, and with only one time point (6 h). In contrast, this study used four different antibiotics (gentamicin, ampicillin, norflorxacin, and trimethoprim), and each antibiotic at different time points, which has never been done before. Interestingly, we found that different persister genes are of varying importance in persistence depending on the length of antibiotic exposure and the type of antibiotics (**Table 1**, **Figures 1–4**). These findings suggest that the persistence phenomenon is not a fixed feature but rather is hierarchical and dynamic in nature, which is consistent with our previous study on the persister gene *phoU* (Li and Zhang, 2007). Our studies confirm and extend the current understanding of persister mechanisms.

In our study, mutants showed a dramatic decrease in persistence at different time points: the decrease in persistence of some mutants occurred at an earlier time point (0.5 h for gentamicin or 4 h for ampicillin and norflorxacin or 3 days for trimethoprim), while some displayed defects at later time points (2 h for gentamicin, 24 h for ampicillin and norflorxacin, or 7 days for trimethoprim). For example, upon exposure to ampicillin for 4 h, *recA*, *lon*, *oxyR*, *phoU*, *dnaK*, and *mqsR* mutants showed the most significant defects, whereas the *relA* mutant responded to this stress with significant defects only after 8 h (**Figure 1**). Similarly, upon norfloxacin exposure, the number of Δ*dnaK* mutant bacteria dropped sharply at 2 h. The number of Δ*recA* and Δ*tisAB* mutants decreased dramatically at 4 h, another nine mutants exhibited a significant decrease in persistence at 24 h (**Figure 2**). These findings may imply different genes are preferentially important at different times for maintaining persister survival. The results of this study support the notion that persister genes are not created equal and can be divided into those involved in shallow persistence, intermediate-level persistence, and deep persistence (Ma et al., 2010), as expressed in the Yin-Yang model (Zhang, 2014).

When the exposure time was extended to 24 h (2 h for gentamicin and 7 days for trimethoprim), the number of persisters of 14 single-deletion mutants (*oxyR*, *dnaK*, *recA*, *relE*, *relA*, *lon*, *glpD*, *mqsR*, *phoU*, *sucB*, *rpoS*, *smpB*, *tisAB*, and *clpB*) involved in multiple pathways all dropped below the detection limit (**Figures 1–4**), indicating that genes and pathways able to affect the persister formation and survival constitute a network, in which components may interact with each other. These results again confirm the redundancy in persister genes or pathways, suggesting that bacterial persistence requires coordination of multiple genes and pathways involved in sensing, stress response/survival, energy production, and DNA repair.

The bi-phasic killing pattern is commonly done on growing culture to demonstrate the persister phenomenon. However, persister assays can also be done with non-growing stationary phase cultures, which can show more clearly persistence phenotype of the persisters and may not show the bi-phasic killing curve. We used stationary phase cultures with a range of different antibiotic exposure time points including early time points which are mostly used in persister studies as well as later time points which are not often used but are important to demonstrate deep persister genes. If we only used early time points as done in most studies, we would miss the effect of a given persister gene or be mistaken that there is no difference in persister levels when a difference indeed exists if later time points are used. Also, we chose the antibiotic exposure timepoints according to the previous work of Tenson and colleagues (Luidalepp et al., 2011) and our group (Li and Zhang, 2007). In fact, when Bigger first gave the term “persister,” he used a long antibiotic exposure time ranging from 1 day to 3 days (Bigger, 1944). Thus, the use of prolonged time points with stationary phase cultures is justified and is in fact important in this study to rank the persister genes in a way that is not previously done. An important difference between this study and the previous studies (Hansen et al., 2008; Dorr et al., 2010) is that we used a range of antibiotic exposure times including an extended antibiotic exposure time of up to 24 h (2 h for gentamicin and 7 days for trimethoprim) in order to examine the ‘deep persisters’ (Ma et al., 2010). Compared to mutants with a minor or “shallow” persistence phenotype (about 10-fold drop in persisters compared with the wild-type strain) observed in the previous study with a shorter time antibiotic exposure time of 5–6 h (Hansen et al., 2008), the persister phenotype was more distinguishable with larger differences in the number of persisters between the mutants and the parent strain in our study with extended exposure times. More importantly, it allows the demonstration of the varying importance of different individual persister genes at different times of drug exposure and the dynamic nature of persisters.

It is interesting to note that significant persistence defects in persister gene mutants was observed even with the bacteriostatic sulfa drug trimethoprim. The *uvrA*, *relA*, *clpB*, *oxyR*, and *sucB* mutants decreased about 3–1000 fold at 3 days, whereas s*mpB*, *dnaK*, *ssrA*, and *mqsR* mutants could be ranked at 5 days. A hierarchy of importance of persistence genes is probably correlated with the adaptation mechanisms of persisters to respond to changes in the environment. These findings are consistent with and support the notion that persisters may consist of different subpopulations of varying hierarchy in continuum (Zhang, 2014). The late persister genes may cooperate with the earlier persister genes to facilitate transition from the shallow to deep persistence in a subpopulation of persisters. It is also likely that they may work in different subpopulations at different times. Moreover, given that trimethoprim is known to inhibit folic acid synthesis, which is essential for synthesis of thymidine triphosphate (dTTP) in bacteria, defects in dTTP synthesis could cause thymine-less death in bacteria (Itsko and Schaaper, 2014), and underlie the increased susceptibility of the mutants to even bacteriostatic sulfa drugs. The significant bactericidal activity of trimethoprim against the above mutants suggests that sulfa drugs could be critical for persister bacteria lacking certain functional pathways, such as stringent response, SOS response, and signaling pathways. The underlying mechanism needs further investigation.

Previous studies have mainly examined the persister genes using a single antibiotic and often single time. Therefore, it is hard to differentiate the most important genes (pathways) and ranked them accurately. To avoid the arbitary ranking of the persister genes, we used four different antibiotics and over different time points. This will insure that the ranking is done in a more precise manner. Our ranking under various antibiotic exposures over time found that *oxyR* (oxidative stress pathway), *dnaK, clpB, and rpoS* (global regulator), *relA* (stringent response), *sucB* (energy production), *mqsR* (toxin–antitoxin modules), and *recA* (SOS response) seem to be prominent genes in this study according to the total scores used to more clearly rank the relative importance of the persister genes (**Table 1**). The eight mutants showed more significant reductions in persistence than other mutants at almost all the time points in exposures to more than two different antibiotics.

The results obtained using different classes of antibiotics indicate that aminoglycoside antibiotic gentamicin can lead to a very low persister level or sterilization of wild-type bacterial cultures, whereas ampicillin, norfloxacin, and especially trimethoprim, leave a detectable fraction of persisters after a 24-h treatment (7 days for trimethoprim). One possibility is that, gentamicin besides inducing misreading in protein synthesis also targets trans-translation pathway (Konno et al., 2004) that has been shown to be involved in persistence (Li et al., 2013) such that gentamicin kills persister bacteria more effectively than other aminoglycoside antibiotics such as streptomycin and hygromycin B that do not inhibit trans-translation (Konno et al., 2004). In contrast to other knockout mutants tested, the deletion mutant of *rpoS* displayed a defect in persistence to gentamicin, but a higher persistence phenotype than wild-type strain in exposure to ampicillin or norfloxacin. Our data are compatible with the previous observation (Hong et al., 2012; Wang et al., 2014) that loss of RpoS renders stationary-phase *E. coli* more sensitive to gentamicin by generating more ROS to enhance oxidative stress, whereas compensatory mutations may have occurred in the RpoS mutant induced by ampicillin and norfloxacin, which led to a higher persistence phenotype. Future studies are needed to address this possibility. Thus, a potential limitation of the study on persister gene deletion is the possible compensation by other genes that may mask the role and, therefore, compromise the study of persister genes under different conditions. Future studies using targeted point mutations in candidate persister genes to avoid compensatory mutations or the polar effects of gene deletions may help to address the limitation of the gene deletion approach.

It is worth noting that the persister levels vary according to laboratory conditions, age of inoculum, specific environment or models used in the study. Hence, the media, cultivation conditions, *E. coli* strain of different genetic background, and viability detection methods used in our study may not lead to results directly comparable to previous studies. Furthermore, data acquired from *in vitro* studies should be evaluated in animal models that imitate human infections. Although persisters *in vitro* are not the same as persisters *in vivo* due to differences in the environments that the bacteria reside in and the presence or absence of antibiotic exposure, the *in vitro* persisters may share some common features of *in vivo* persisters and should have value in persister studies as surrogates of *in vivo* persisters (Zhang, 2014). Future studies are needed to validate the findings of this *in vitro* study in animal models.

## Conclusion

The present study extends our concept of bacterial persisters by demonstrating the varying hierarchy of importance of persister genes or pathways. Our study indicates that different persister genes play key roles at different times and according to different antibiotics. Our data also provide evidence for the notion that persister genes could be divided into shallow persistence and deep persistence genes. The identified key persister genes may serve as targets for development of new drugs against persisters for more effective treatment of persistent bacterial infections.

## Author Contributions

YZ and WZ designed the experiments; NW, LH, PC, WW, YY, and SL performed the gene knockout experiments; NW, SL, TX, SZ, and JW performed the ranking experiments. NW, PC, YY, and SZ analyzed the data. NW wrote the manuscript.

## Conflict of Interest Statement

The authors declare that the research was conducted in the absence of any commercial or financial relationships that could be construed as a potential conflict of interest.

## References

[B1] Amato S. M. Fazen C. H. Henry T. C. Mok W. W. Orman M. A. Sandvik E. L. (2014). The role of metabolism in bacterial persistence. *Front. Microbiol.* 5:70 10.3389/fmicb.2014.00070 PMC3939429 24624123

[B2] Balaban N. Q. Merrin J. Chait R. Kowalik L. Leibler S. (2004). Bacterial persistence as a phenotypic switch. *Science* 305 1622–1625. 10.1126/science.1099390 15308767

[B3] Bigger J. W. (1944). Treatment of staphylococcal infections with penicillin BY Intermittent Sterilisation. *Lancet* 244 497–500. 10.1016/S0140-6736(00)74210-3

[B4] Blango M. G. Mulvey M. A. (2010). Persistence of uropathogenic *Escherichia coli* in the face of multiple antibiotics. *Antimicrob. Agents Chemother.* 54 1855–1863. 10.1128/AAC.00014-10 20231390 PMC2863638

[B5] Buts L. Lah J. Dao-Thi M. H. Wyns L. Loris R. (2005). Toxin-antitoxin modules as bacterial metabolic stress managers. *Trends Biochem. Sci.* 30 672–679. 10.1016/j.tibs.2005.10.004 16257530

[B6] Datsenko K. A. Wanner B. L. (2000). One-step inactivation of chromosomal genes in *Escherichia coli* K-12 using PCR products. *Proc. Natl. Acad. Sci. U.S.A.* 97 6640–6645. 10.1073/pnas.120163297 10829079 PMC18686

[B7] Debbia E. A. Roveta S. Schito A. M. Gualco L. Marchese A. (2001). Antibiotic persistence: the role of spontaneous DNA repair response. *Microb. Drug Resist. (Larchmont, N.Y.)* 7 335–342. 10.1089/10766290152773347 11822773

[B8] Dorr T. Lewis K. Vulic M. (2009). SOS response induces persistence to fluoroquinolones in *Escherichia coli*. *PLoS Genet.* 5:e1000760 10.1371/journal.pgen.1000760 PMC2780357 20011100

[B9] Dorr T. Vulic M. Lewis K. (2010). Ciprofloxacin causes persister formation by inducing the TisB toxin in *Escherichia coli*. *PLoS Biol.* 8:e1000317 10.1371/journal.pbio.1000317 PMC2826370 20186264

[B10] Falla T. J. Chopra I. (1998). Joint tolerance to beta-lactam and fluoroquinolone antibiotics in *Escherichia coli* results from overexpression of hipA. *Antimicrob. Agents Chemother.* 42 3282–3284. 9835528 10.1128/aac.42.12.3282 PMC106036

[B11] Gerard F. Dri A. M. Moreau P. L. (1999). Role of *Escherichia coli* RpoS, LexA and H-NS global regulators in metabolism and survival under aerobic, phosphate-starvation conditions. *Microbiology* 145(Pt 7), 1547–1562. 10439394 10.1099/13500872-145-7-1547

[B12] Girgis H. S. Harris K. Tavazoie S. (2012). Large mutational target size for rapid emergence of bacterial persistence. *Proc. Natl. Acad. Sci. U.S.A.* 109 12740–12745. 10.1073/pnas.1205124109 22802628 PMC3411964

[B13] Hansen S. Lewis K. Vulic M. (2008). Role of global regulators and nucleotide metabolism in antibiotic tolerance in *Escherichia coli*. *Antimicrob. Agents Chemother.* 52 2718–2726. 10.1128/AAC.00144-08 18519731 PMC2493092

[B14] Hengge-Aronis R. (1996). Back to log phase: sigma S as a global regulator in the osmotic control of gene expression in *Escherichia coli*. *Mol. Microbiol.* 21 887–893. 10.1046/j.1365-2958.1996.511405.x 8885260

[B15] Hobby G. L. Meyer K. Chaffee E. (1942). Observations on the mechanism of action of penicillin. *Proc. Soc. Exp. Biol. Med.* 50 281–285. 10.3181/00379727-50-13773

[B16] Hong S. H. Wang X. O’Connor H. F. Benedik M. J. Wood T. K. (2012). Bacterial persistence increases as environmental fitness decreases. *Microb. Biotechnol.* 5 509–522. 10.1111/j.1751-7915.2011.00327.x 22221537 PMC3323757

[B17] Itsko M. Schaaper R. M. (2014). dGTP starvation in *Escherichia coli* provides new insights into the thymineless-death phenomenon. *PLoS Genet.* 10:e1004310 10.1371/journal.pgen.1004310 PMC4014421 24810600

[B18] Kaspy I. Rotem E. Weiss N. Ronin I. Balaban N. Q. Glaser G. (2013). HipA-mediated antibiotic persistence via phosphorylation of the glutamyl-tRNA-synthetase. *Nat. Commun.* 4 3001 10.1038/ncomms4001 24343429

[B19] Keren I. Shah D. Spoering A. Kaldalu N. Lewis K. (2004). Specialized persister cells and the mechanism of multidrug tolerance in *Escherichia coli*. *J. Bacteriol.* 186 8172–8180. 10.1128/JB.186.24.8172-8180.2004 15576765 PMC532439

[B20] Kim Y. Wang X. Zhang X. S. Grigoriu S. Page R. Peti W. (2010). *Escherichia coli* toxin/antitoxin pair MqsR/MqsA regulate toxin CspD. *Environ. Microbiol.* 12 1105–1121. 10.1111/j.1462-2920.2009.02147.x 20105222 PMC3980499

[B21] Kint C. I. Verstraeten N. Fauvart M. Michiels J. (2012). New-found fundamentals of bacterial persistence. *Trends Microbiol.* 20 577–585. 10.1016/j.tim.2012.08.009 22959615

[B22] Konno T. Takahashi T. Kurita D. Muto A. Himeno H. (2004). A minimum structure of aminoglycosides that causes an initiation shift of trans-translation. *Nucleic Acids Res.* 32 4119–4126. 10.1093/nar/gkh750 15295039 PMC514373

[B23] Korch S. B. Henderson T. A. Hill T. M. (2003). Characterization of the hipA7 allele of *Escherichia coli* and evidence that high persistence is governed by (p)ppGpp synthesis. *Mol. Microbiol.* 50 1199–1213. 10.1046/j.1365-2958.2003.03779.x 14622409

[B24] Levin B. R. Concepcion-Acevedo J. Udekwu K. I. (2014). Persistence: a copacetic and parsimonious hypothesis for the existence of non-inherited resistance to antibiotics. *Curr. Opin. Microbiol.* 21C, 18–21. 10.1016/j.mib.2014.06.016 25090240 PMC4253300

[B25] Lewis K. (2010). Persister cells. *Annu. Rev. Microbiol.* 64 357–372. 10.1146/annurev.micro.112408.134306 20528688

[B26] Li J. Ji L. Shi W. Xie J. Zhang Y. (2013). Trans-translation mediates tolerance to multiple antibiotics and stresses in *Escherichia coli*. *J. Antimicrob. Chemother.* 68 2477–2481. 10.1093/jac/dkt231 23812681 PMC3797643

[B27] Li Y. Zhang Y. (2007). PhoU is a persistence switch involved in persister formation and tolerance to multiple antibiotics and stresses in *Escherichia coli*. *Antimicrob. Agents Chemother.* 51 2092–2099. 10.1128/AAC.00052-07 17420206 PMC1891003

[B28] Luidalepp H. Joers A. Kaldalu N. Tenson T. (2011). Age of inoculum strongly influences persister frequency and can mask effects of mutations implicated in altered persistence. *J. Bacteriol.* 193 3598–3605. 10.1128/JB.00085-11 21602347 PMC3133311

[B29] Ma C. Sim S. Shi W. Du L. Xing D. Zhang Y. (2010). Energy production genes sucB and ubiF are involved in persister survival and tolerance to multiple antibiotics and stresses in *Escherichia coli*. *FEMS Microbiol. Lett.* 303 33–40. 10.1111/j.1574-6968.2009.01857.x 20041955

[B30] Maisonneuve E. Shakespeare L. J. Jorgensen M. G. Gerdes K. (2011). Bacterial persistence by RNA endonucleases. *Proc. Natl. Acad. Sci. U.S.A.* 108 13206–13211. 10.1073/pnas.1100186108 21788497 PMC3156201

[B31] Matsuoka Y. Shimizu K. (2011). Metabolic regulation in *Escherichia coli* in response to culture environments via global regulators. *Biotechnol. J.* 6 1330–1341. 10.1002/biot.201000447 21433292

[B32] Michel B. (2005). After 30 years of study, the bacterial SOS response still surprises us. *PLoS Biol.* 3:e255 10.1371/journal.pbio.0030255 PMC1174825 16000023

[B33] Moyed H. S. Bertrand K. P. (1983). hipA, a newly recognized gene of *Escherichia coli* K-12 that affects frequency of persistence after inhibition of murein synthesis. *J. Bacteriol.* 155 768–775. 6348026 10.1128/jb.155.2.768-775.1983 PMC217749

[B34] Muﬄer A. Traulsen D. D. Lange R. Hengge-Aronis R. (1996). Posttranscriptional osmotic regulation of the sigma(s) subunit of RNA polymerase in *Escherichia coli*. *J. Bacteriol.* 178 1607–1613. 8626288 10.1128/jb.178.6.1607-1613.1996 PMC177845

[B35] Shi W. Zhang X. Jiang X. Yuan H. Lee J. S. Barry C. E. (2011). Pyrazinamide inhibits trans-translation in *Mycobacterium tuberculosis*. *Science* 333 1630–1632. 10.1126/science.1208813 21835980 PMC3502614

[B36] Singh R. Ray P. Das A. Sharma M. (2009). Role of persisters and small-colony variants in antibiotic resistance of planktonic and biofilm-associated *Staphylococcus aureus*: an in vitro study. *J. Med. Microbiol.* 58 1067–1073. 10.1099/jmm.0.009720-0 19528167

[B37] Singh V. K. Utaida S. Jackson L. S. Jayaswal R. K. Wilkinson B. J. Chamberlain N. R. (2007). Role for dnaK locus in tolerance of multiple stresses in *Staphylococcus aureus*. *Microbiology* 153 3162–3173. 10.1099/mic.0.2007/009506-0 17768259

[B38] Squires C. L. Pedersen S. Ross B. M. Squires C. (1991). ClpB is the *Escherichia coli* heat shock protein F84.1. *J. Bacteriol.* 173 4254–4262. 2066329 10.1128/jb.173.14.4254-4262.1991 PMC208084

[B39] Straus D. Walter W. Gross C. A. (1990). DnaK, DnaJ, and GrpE heat shock proteins negatively regulate heat shock gene expression by controlling the synthesis and stability of sigma 32. *Genes Dev.* 4 2202–2209. 2269429 10.1101/gad.4.12a.2202

[B40] Szabo A. Langer T. Schroder H. Flanagan J. Bukau B. Hartl F. U. (1994). The ATP hydrolysis-dependent reaction cycle of the *Escherichia coli* Hsp70 system DnaK, DnaJ, and GrpE. *Proc. Natl. Acad. Sci. U.S.A.* 91 10345–10349. 10.1073/pnas.91.22.10345 7937953 PMC45016

[B41] Tashiro Y. Kawata K. Taniuchi A. Kakinuma K. May T. Okabe S. (2012). RelE-mediated dormancy is enhanced at high cell density in *Escherichia coli*. *J. Bacteriol.* 194 1169–1176. 10.1128/JB.06628-11 22210768 PMC3294780

[B42] Tilly K. McKittrick N. Zylicz M. Georgopoulos C. (1983). The dnaK protein modulates the heat-shock response of *Escherichia coli*. *Cell* 34 641–646. 10.1016/0092-8674(83)90396-3 6311435

[B43] Vazquez-Laslop N. Lee H. Neyfakh A. A. (2006). Increased persistence in *Escherichia coli* caused by controlled expression of toxins or other unrelated proteins. *J. Bacteriol.* 188 3494–3497. 10.1128/JB.188.10.3494-3497.2006 16672603 PMC1482871

[B44] Vega N. M. Allison K. R. Khalil A. S. Collins J. J. (2012). Signaling-mediated bacterial persister formation. *Nat. Chem. Biol.* 8 431–433. 10.1038/nchembio.915 22426114 PMC3329571

[B45] Wang J. H. Singh R. Benoit M. Keyhan M. Sylvester M. Hsieh M. (2014). Sigma S-dependent antioxidant defense protects stationary-phase *Escherichia coli* against the bactericidal antibiotic gentamicin. *Antimicrob. Agents Chemother.* 58 5964–5975. 10.1128/AAC.03683-14 25070093 PMC4187989

[B46] Wang X. Lord D. M. Hong S. H. Peti W. Benedik M. J. Page R. (2013). Type II toxin/antitoxin MqsR/MqsA controls type V toxin/antitoxin GhoT/GhoS. *Environ. Microbiol.* 15 1734–1744. 10.1111/1462-2920.12063 23289863 PMC3620836

[B47] Wang X. Wood T. K. (2011). Toxin-antitoxin systems influence biofilm and persister cell formation and the general stress response. *Appl. Environ. Microbiol.* 77 5577–5583. 10.1128/AEM.05068-11 21685157 PMC3165247

[B48] Yeh J. I. Chinte U. Du S. (2008). Structure of glycerol-3-phosphate dehydrogenase, an essential monotopic membrane enzyme involved in respiration and metabolism. *Proc. Natl. Acad. Sci. U.S.A.* 105 3280–3285. 10.1073/pnas.0712331105 18296637 PMC2265192

[B49] Zhang Y. (2014). Persisters, persistent infections and the Yin-Yang model. *Emerg. Microbes Infect.* 3 e3 10.1038/emi.2014.3 PMC3913823 26038493

[B50] Zhang Y. Yew W. W. Barer M. R. (2012). Targeting persisters for tuberculosis control. *Antimicrob. Agents Chemother.* 56 2223–2230. 10.1128/AAC.06288-11 22391538 PMC3346619

