# Supplementary material for: Ranking of persister genes in the same Escherichia coli genetic background demonstrates varying importance of individual persister genes in tolerance to different antibiotics
Source: Front Microbiol. 2015 Sep 30;6:1003. doi: 10.3389/fmicb.2015.01003 (PMC4588708; doi:10.3389/fmicb.2015.01003)
Supplement: Supplementary file 1 [file Supplementary_Material.DOC]

***Supplementary Material***

**Ranking of persister genes in the same *Escherichia coli* genetic background demonstrates varying importance of individual persister genes in tolerance to antibiotics**

Nan Wu^1^, Lei He^1^, Peng Cui^1^, Wenjie Wang^1^, Youhua Yuan^1^, Shuang Liu^1^, Tao Xu^1^, Jing Wu^1^, Wenhong Zhang^1*^ and Ying Zhang^1,2*^

^1^ Key Lab of Molecular Virology, Institute of Medical Microbiology, Department of Infectious Diseases, Huashan Hospital, Fudan University, Shanghai, China

^2^ Department of Molecular Microbiology and Immunology, Bloomberg School of Public Health, Johns Hopkins University, Baltimore, Maryland, USA

***Correspondence:**

Ying Zhang, MD, PhD

Department of Molecular Microbiology and Immunology,

Bloomberg School of Public Health,

Johns Hopkins University,

Baltimore, MD 21205, USA

Email: yzhang@jhsph.edu

**Table S1. Homologous arm primers used for generating gene knockout mutants.**

| **Gene** | **Primer (5’-3’)** |
| --- | --- |
| *glpD* | F: TCAGTCTCAGGCCAGATTGAAATCTGACCTGATCACCTTACGTTAATTTAATGGGAATTAGCCATGGTCC  R:TGTTCGATAACGAACATTTATGAGCTTTAACGAAAGTGAATGAGGGCAGCGTGTAGGCTGGAGCTGCTTC |
| *relA* | F: GTAGATACAGTATATATCAATCTACATTGTAGATACGAGCAAATTTCGGCATGGGAATTAGCCATGGTCC  R:TAGTTGCGATTTGCCGATTTCGGCAGGTCTGGTCCCTAAAGGAGAGGACGGTGTAGGCTGGAGCTGCTTC |
| *uvrA* | F:GGTGCAACTCTGAAAGGAAAAGGCCGCTCAGAAAGCGGCCTTAACGATTAATGGGAATTAGCCATGGTCC  R:CGGTAGCACCATGCCACCGGGCAAAAAAGCGTTTAATCCGGGAAAGGTGAGTGTAGGCTGGAGCTGCTTC |
| *umuD* | F: CAAGAACAGACTACTGTATATAAAAACAGTATAACTTCAGGCAGATTATTATGGGAATTAGCCATGGTCC  R: CGTCTCACAGCTGGCATAAAACGCGTTTACATCACAGAGGGCAAACATCAGTGTAGGCTGGAGCTGCTTC |
| *lon* | F: CAGTCGTGTCATCTGATTACCTGGCGGAAATTAAACTAAGAGAGAGCTCTATGGGAATTAGCCATGGTCC  R: ATTAGCCTGCCAGCCCTGTTTTTATTAGTGCATTTTGCGCGAGGTCACTAGTGTAGGCTGGAGCTGCTTC |
| *relE* | F: GAACGGCTTCGTAATCCTAAGCCAGTACGTGTGACGCTGGATGAACTCTGATGGGAATTAGCCATGGTCC  R: AGCAGAAGTGTCACCTTCGGTGCGAAACAGAGATGTCATGCTTTGGTTCAGTGTAGGCTGGAGCTGCTTC |
| *smpB* | F: ATTGATATGGGGTGTTTTCGATTTCAGATTACCGATGATTCACGACGCTTATGGGAATTAGCCATGGTCC  R:GGAGAGGGAGGCGCGGTGAGGAACTGGTCAATAATTGGAGTGCAGGTTTAGTGTAGGCTGGAGCTGCTTC |
| *ssrA* | F: TGAGGCTGGTCATGGCGCTCATAAATCTGGTATACTTACCTTTACACATTATGGGAATTAGCCATGGTCC  R: GGCTCTTAGGACTTCATCGGATGACTCTGGTAATCACCGATGGAGAATTTGTGTAGGCTGGAGCTGCTTC |
| *dinJ* | F: ATCCTTTGAATATTGTCCCGAGTATTCAATATCCCTTTGAATCATATTTAATGGGAATTAGCCATGGTCC  R: TTTGAGCTACAATTCAAGCTGAATAAATATACAGCACAGGAGATACCCCAGTGTAGGCTGGAGCTGCTTC |
| *rpoS* | F: AGGCTTTTGCTTGAATGTTCCGTCAAGGGATCACGGGTAGGAGCCACCTTATGGGAATTAGCCATGGTCC  R: ACAGAAAAGGCCAGCCTCGCTTGAGACTGGCCTTTCTGACAGATGCTTACGTGTAGGCTGGAGCTGCTTC |
| *tnaA* | F: AGGGTAAGAGAGTGGCTAACATCCTTATAGCCACTCTGTAGTATTAATTAATGGGAATTAGCCATGGTCC  R: TGTAATATTCACAGGGATCACTGTAATTAAAATAAATGAAGGATTATGTAGTGTAGGCTGGAGCTGCTTC |
| *pspF* | F: AATAAAGCATTCACGCCGCATCCGGCAAGTTGTATTGCTCAACTTCGCTAATGGGAATTAGCCATGGTCC  R: ACATGCCAGGATGAGTTAGCGAATTACACTAACAAGTGGCGAATTTCATCGTGTAGGCTGGAGCTGCTTC |
| *mqsR* | F: CTGTAATTAACCTTTTAGGTTATAACTAAAGTAACAGGGAGGCGGGGGTTATGGGAATTAGCCATGGTCC  R: CTTTAATGCCAGAAACCATTTCTCCCTGGTGGCAAACCGGACATTTCATAGTGTAGGCTGGAGCTGCTTC |
| *clpB* | F: TGACCTCATTTAATCTCCAGTAGCAACTTTGATCCGTTATGGGAGGAGTTATGGGAATTAGCCATGGTCC  R: TCCGTCTAACTTATAGACAAAAACGAGCCCCGAAGGGCTCGTTTTATCATGTGTAGGCTGGAGCTGCTTC |
| *phoU* | F: CAAACAGAAGACTACATCACCGGTCGTTACGGTTGATTCAGGAGTGCGTTATGGGAATTAGCCATGGTCC  R: TTATATATAACAAATCCCAATAATTAAGTTATTGGGATTTGTCTGGTGAAGTGTAGGCTGGAGCTGCTTC |
| *hipA* | F:GCGAAAAATGCCTCGCCAGAATCAACAGAACAGCAAAATCTGGAGTGGTAATGGGAATTAGCCATGGTCC  R: GTTGGCGGTCATGATTGTCATGCTCATTAACAATGACCAAACCCCATATC GTGTAGGCTGGAGCTGCTTC |
| *recA* | F: GTAGATACAGTATATATCAATCTACATTGTAGATACGAGCAAATTTCGGCATGGGAATTAGCCATGGTCC  R: TAGTTGCGATTTGCCGATTTCGGCAGGTCTGGTCCCTAAAGGAGAGGACGGTGTAGGCTGGAGCTGCTTC |
| *tisAB* | F: GAAACGGGTGGTGCCGTCAGCGCCTTAACCCCGCGTGAGCACACTGTGTTATGGGAATTAGCCATGGTCC  R: ATAAAAGGGGAGCGGTTTCCCGCTCCCCTTTGGTGCGACTTGAATCTGAAGTGTAGGCTGGAGCTGCTTC |
| *sucB* | F:CAAGATCTGGTTAATGACGCGCTGAACGTCGAATAAATAAAGGATACACAATGGGAATTAGCCATGGTCC  R: GGCGATAATGCCTTATCCGGTCTACAGTGCAGGTGAAACTTAAACTACTAGTGTAGGCTGGAGCTGCTTC |
| *oxyR* | F: ACTACCCGACGATGGCGGAAGCCTATCGGGTAGCTGCGTTAAACGGTTTAATGGGAATTAGCCATGGTCC  R: TTGCTATTCTACCTATCGCCATGAACTATCGTGGCGATGGAGGATGGATAGTGTAGGCTGGAGCTGCTTC |
| *dnaK* | F: TTACAGACTCACAACCACATGATGACCGAATATATAGTGGAGACGTTTAGATGGGAATTAGCCATGGTCC  R: AAATTCCCCTTCGCCCGTGTCAGTATAATTACCCGTTTATAGGGCGATTAGTGTAGGCTGGAGCTGCTTC |

**Table S2. Gene and Pathway ranking depends on exposure to different antibiotics at the first time point ***

***** The first timepoint for ampicillin and norfloxacin is 4 h, for gentamicin at 0.5 h, and for trimethoprim at 3 days;

|  | **Ampicillin** | | | **Norfloxacin** | | | **Gentamicin** | | | **Trimethoprim** | | |
| --- | --- | --- | --- | --- | --- | --- | --- | --- | --- | --- | --- | --- |
|  | **Genes** | **Pathways** | **Score** | **Genes** | **Pathways** | **Score** | **Genes** | **Pathways** | **Score** | **Genes** | **Pathways** | **Score** |
| 1 | *recA* | SOS  response | 1 | *dnaK* | Global regulator | 1 | *rpoS* | Global regulator | 1 | *uvrA* | SOS respons | 1 |
| 2 | *lon* | TA  module | 1 | *tisAB* | TA  module | 1 | *sucB* | Energy production | 1 | *relA* | Stringent response | 1 |
| 3 | *oxyR* | Antioxidant defense | 1 | *recA* | SOS response | 1 | *oxyR* | Antioxidant defense | 1 | *clpB* | Global regulator | 1 |
| 4 | *phoU* | Global regulator | 1 | *mqsR* | TA  module | 0 | *phoU* | Global regulator | 0 | *oxyR* | Antioxidant defense | 1 |
| 5 | *dnaK* | Global regulator | 1 | *pspF* | Signaling pathway | 0 | *recA* | SOS response | 0 | *sucB* | Energy production | 1 |
| 6 | *mqsR* | TA  module | 1 | *relA* | Stringent response | 0 | *umuD* | SOS response | 0 | *ssrA* | Trans-translation | 0 |
| 7 | *sucB* | Energy production | 0 | *lon* | TA  module | 0 | *hipA* | TA  module | 0 | *smpB* | Trans-translation | 0 |
| 8 | *glpD* | Energy production | 0 | *clpB* | Global regulator | 0 | *dnaK* | Global regulator | 0 | *relE* | TA  module | 0 |
| 9 | *smpB* | Trans-translation | 0 | *phoU* | Global regulator | 0 | *smpB* | Trans-translation | 0 | *mqsR* | TA  module | 0 |
| 10 | *dinJ* | TA  module | 0 | *hipA* | TA  module | 0 | *lon* | TA  module | 0 | *dnaK* | Global regulator | 0 |
| 11 | *rpoS* | Global regulator | 0 | *tnaA* | Signaling pathway | 0 | *mqsR* | TA  module | 0 | *tnaA* | Signaling pathway | 0 |
| 12 | *umuD* | SOS  response | 0 | *smpB* | Trans-translation | 0 | *ssrA* | Trans-translation | 0 | *hipA* | TA  module | 0 |
| 13 | *relE* | TA  module | 0 | *relE* | TA  module | 0 | *relE* | TA  module | 0 | *phoU* | Global regulator | 0 |
| 14 | *tisAB* | TA  module | 0 | *ssrA* | Trans-translation | 0 | *clpB* | Global regulator | 0 | *glpD* | Energy production | 0 |
| 15 | *pspF* | Signaling pathway | 0 | *umuD* | SOS response | 0 | *tisAB* | TA  module | 0 | *lon* | TA  module | 0 |
| 16 | *clpB* | Global regulator | 0 | *dinJ* | TA  module | 0 | *dinJ* | TA  module | 0 | *tisAB* | TA  module | 0 |
| 17 | *relA* | Stringent response | 0 | *glpD* | Energy production | 0 | *uvrA* | SOS respons | 0 | *dinJ* | TA  module | 0 |
| 18 | *ssrA* | Trans-translation | 0 | *rpoS* | Global regulator | 0 | *tnaA* | Signaling pathway | 0 | *rpoS* | Global regulator | 0 |
| 19 | *uvrA* | SOS  response | 0 | *uvrA* | SOS response | 0 | *pspF* | Signaling pathway | 0 | *umuD* | SOS response | 0 |
| 20 | *tnaA* | Signaling pathway | 0 | *oxyR* | Antioxidant defense | 0 | *relA* | Stringent response | 0 | *recA* | SOS response | 0 |
| 21 | *hipA* | TA  module | 0 | *sucB* | Energy production | 0 | *glpD* | Energy production | 0 | *pspF* | Signaling pathway | 0 |

“1” : significant change compared with control W3110

“0” : no significant change

**Table S3. Gene and Pathway ranking depends on exposure to different antibiotics at the second time point ***

***** The second timepoint for ampicillin and norfloxacin is 8 h, for gentamicin at 1 h, and for trimethoprim at 5 days;

|  | **Ampicillin** | | | **Norfloxacin** | | | **Gentamicin** | | | **Trimethoprim** | | |
| --- | --- | --- | --- | --- | --- | --- | --- | --- | --- | --- | --- | --- |
|  | **Genes** | **Pathways** | **Score** | **Genes** | **Pathways** | **Score** | **Genes** | **Pathways** | **Score** | **Genes** | **Pathways** | **Score** |
| 1 | *oxyR* | Antioxidant defense | 1 | *dnaK* | Global regulator | 1 | *rpoS* | Global regulator | 1 | *relA* | Stringent response | 1 |
| 2 | *dnaK* | Global regulator | 1 | *relA* | Stringent response | 1 | *smpB* | Trans-translation | 1 | *clpB* | Global regulator | 1 |
| 3 | *recA* | SOS  response | 1 | *recA* | SOS response | 1 | *sucB* | Energy production | 1 | *sucB* | Energy production | 1 |
| 4 | *lon* | TA  module | 1 | *tisAB* | TA  module | 1 | *hipA* | TA  module | 0 | *oxyR* | Antioxidant defense | 1 |
| 5 | *relA* | Stringent response | 1 | *relE* | TA  module | 1 | *relA* | Stringent response | 0 | *uvrA* | SOS response | 1 |
| 6 | *glpD* | Energy production | 1 | *clpB* | Global regulator | 1 | *oxyR* | Antioxidant defense | 0 | *smpB* | Trans-translation | 1 |
| 7 | *mqsR* | TA  module | 1 | *oxyR* | Antioxidant defense | 1 | *recA* | SOS response | 0 | *dnaK* | Global regulator | 1 |
| 8 | *phoU* | Global regulator | 1 | *lon* | TA  module | 1 | *relE* | TA  module | 0 | *ssrA* | Trans-translation | 1 |
| 9 | *sucB* | Energy production | 1 | *glpD* | Energy production | 1 | *umuD* | SOS response | 0 | *mqsR* | TA  module | 1 |
| 10 | *tnaA* | Signaling pathway | 0 | *mqsR* | TA  module | 0 | *mqsR* | TA  module | 0 | *tnaA* | Signaling pathway | 0 |
| 11 | *clpB* | Global regulator | 0 | *smpB* | Trans-translation | 0 | *lon* | TA  module | 0 | *relE* | TA  module | 0 |
| 12 | *ssrA* | Trans-translation | 0 | *umuD* | SOS response | 0 | *tnaA* | Signaling pathway | 0 | *glpD* | Energy production | 0 |
| 13 | *dinJ* | TA  module | 0 | *tnaA* | Signaling pathway | 0 | *tisAB* | TA  module | 0 | *umuD* | SOS response | 0 |
| 14 | *tisAB* | TA  module | 0 | *pspF* | Signaling pathway | 0 | *dnaK* | Global regulator | 0 | *lon* | TA  module | 0 |
| 15 | *pspF* | Signaling pathway | 0 | *ssrA* | Trans-translation | 0 | *phoU* | Global regulator | 0 | *recA* | SOS response | 0 |
| 16 | *uvrA* | SOS  response | 0 | *hipA* | TA  module | 0 | *glpD* | Energy production | 0 | *phoU* | Global regulator | 0 |
| 17 | *hipA* | TA  module | 0 | *sucB* | Energy production | 0 | *ssrA* | Trans-translation | 0 | *hipA* | TA  module | 0 |
| 18 | *smpB* | Trans-translation | 0 | *dinJ* | TA  module | 0 | *clpB* | Global regulator | 0 | *tisAB* | TA  module | 0 |
| 19 | *relE* | TA  module | 0 | *uvrA* | SOS response | 0 | *uvrA* | SOS response | 0 | *dinJ* | TA  module | 0 |
| 20 | *umuD* | SOS  response | 0 | *phoU* | Global regulator | 0 | *pspF* | Signaling pathway | 0 | *pspF* | Signaling pathway | 0 |
| 21 | *rpoS* | Global regulator | 1 | *rpoS* | Global regulator | 1 | *dinJ* | TA  module | 0 | *rpoS* | Global regulator | 0 |

“1” : significant change compared with control W3110

“0” : no significant change.

**Table S4. Gene and Pathway ranking depends on exposure to different antibiotics at the last time point ***

***** The last timepoint for ampicillin and norfloxacin is 24 h, for gentamicin at 2 h, and for trimethoprim at 7 days;

|  | **Ampicillin** | | | **Norfloxacin** | | | **Gentamicin** | | | **Trimethoprim** | | |
| --- | --- | --- | --- | --- | --- | --- | --- | --- | --- | --- | --- | --- |
|  | **Genes** | **Pathways** | **Score** | **Genes** | **Pathways** | **Score** | **Genes** | **Pathways** | **Score** | **Genes** | **Pathways** | **Score** |
| 1 | *oxyR* | Antioxidant defense | 1 | *dnaK* | Global regulator | 1 | *rpoS* | Global regulator | 1 | *relA* | Stringent response | 1 |
| 2 | *dnaK* | Global regulator | 1 | *relA* | Stringent response | 1 | *smpB* | Trans-translation | 1 | *clpB* | Global regulator | 1 |
| 3 | *recA* | SOS response | 1 | *oxyR* | Antioxidant defense | 1 | *phoU* | Global regulator | 1 | *dnaK* | Global regulator | 1 |
| 4 | *lon* | TA  module | 1 | *clpB* | Global regulator | 1 | *oxyR* | Antioxidant defense | 1 | *sucB* | Energy production | 1 |
| 5 | *relA* | Stringent response | 1 | *recA* | SOS response | 1 | *dnaK* | Global regulator | 1 | *oxyR* | Antioxidant defense | 1 |
| 6 | *glpD* | Energy production | 1 | *relE* | TA  module | 1 | *relA* | Stringent response | 1 | *uvrA* | SOS response | 1 |
| 7 | *mqsR* | TA  module | 1 | *glpD* | Energy production | 1 | *relE* | TA  module | 1 | *smpB* | Trans-translation | 1 |
| 8 | *phoU* | Global regulator | 1 | *lon* | TA  module | 1 | *sucB* | Energy production | 1 | *ssrA* | Trans-translation | 1 |
| 9 | *sucB* | Energy production | 1 | *tisAB* | TA  module | 1 | *ssrA* | Trans-translation | 1 | *mqsR* | TA  module | 1 |
| 10 | *relE* | TA  module | 1 | *mqsR* | TA  module | 1 | *hipA* | TA  module | 1 | *tnaA* | Signaling pathway | 0 |
| 11 | *clpB* | Global regulator | 1 | *umuD* | SOS response | 1 | *umuD* | SOS response | 0 | *umuD* | SOS response | 0 |
| 12 | *tnaA* | Signaling pathway | 0 | *tnaA* | Signaling pathway | 1 | *recA* | SOS response | 0 | *glpD* | Energy production | 0 |
| 13 | *uvrA* | SOS response | 0 | *pspF* | Signaling pathway | 0 | *lon* | TA  module | 0 | *phoU* | Global regulator | 0 |
| 14 | *hipA* | TA  module | 0 | *smpB* | Trans-translation | 0 | *mqsR* | TA  module | 0 | *relE* | TA  module | 0 |
| 15 | *ssrA* | Trans-translation | 0 | *phoU* | Global regulator | 0 | *tnaA* | Signaling pathway | 0 | *tisAB* | TA  module | 0 |
| 16 | *dinJ* | TA  module | 0 | *hipA* | TA  module | 0 | *tisAB* | TA  module | 0 | *recA* | SOS response | 0 |
| 17 | *smpB* | Trans-translation | 0 | *sucB* | Energy production | 0 | *dinJ* | TA  module | 0 | *hipA* | TA  module | 0 |
| 18 | *umuD* | SOS response | 0 | *uvrA* | SOS response | 0 | *glpD* | Energy production | 0 | *pspF* | Signaling pathway | 0 |
| 19 | *tisAB* | TA  module | 0 | *ssrA* | Trans-translation | 0 | *clpB* | Global regulator | 0 | *lon* | TA  module | 0 |
| 20 | *pspF* | Signaling pathway | 0 | *dinJ* | TA  module | 0 | *pspF* | Signaling pathway | 0 | *dinJ* | TA  module | 0 |
| 21 | *rpoS* | Global regulator | 1 | *rpoS* | Global regulator | 1 | *uvrA* | SOS response | 0 | *rpoS* | Global regulator | 0 |

“1” : significant change compared with control W3110

“0” : no significant change.
